# Establishment and Analysis of an Individualized Immune-Related Gene Signature for the Prognosis of Gastric Cancer

**DOI:** 10.3389/fsurg.2022.829237

**Published:** 2022-01-31

**Authors:** Mengying Li, Wei Cao, Bingqian Huang, Zhipeng Zhu, Yaxin Chen, Jiawei Zhang, Guodong Cao, Bo Chen

**Affiliations:** ^1^Department of General Surgery, First Affiliated Hospital of Anhui Medical University, Hefei, China; ^2^Department of Clinical Medicine, Anhui Medical University, Hefei, China

**Keywords:** gastric cancer, immune gene, prognosis, computational biology, biomarker

## Abstract

A growing number of studies have shown that immunity plays an important clinical role in the process of gastric cancer (GC). The purpose of this study was to explore the function of differentially expressed immune-related genes (DEIRGs) of GC, and construct a gene signature to predict the overall survival (OS) of patients. Gene expression profiles and clinical data of GC patients were downloaded from TCGA and GEO databases. Combined with immune-related genes (IRGs) downloaded from the ImmPort database, 357 DEIRGs in GC tissues and adjacent tissues were identified. Based on the analysis of Lasso and Cox in the training set, a prognostic risk scoring model consisting of 9 (RBP7, DES, CCR1, PNOC, SPP1, VIP, TNFRSF12A, TUBB3, PRKCG) DEIRGs was obtained. Functional analysis revealed that model genes may participate in the formation and development of tumor cells by affecting the function of cell gap junction intercellular communication (GJJC). According to the model score, the samples were divided into high-risk and low-risk groups. In multivariate Cox regression analysis, the risk score was an independent prognostic factor (HR = 1.674, 95% CI = 1.470–1.907, *P* < 0.001). Survival analysis showed that the OS of high-risk GC patients was significantly lower than that of low-risk GC patients (*P* < 0.001). The area under the receiver operating characteristic curve (ROC) of the model was greater than other clinical indicators when verified in various data sets, confirming that the prediction model has a reliable accuracy. In conclusion, this study has explored the biological functions of DEIRGs in GC and discovered novel gene targets for the treatment of GC. The constructed prognostic gene signature is helpful for clinicians to determine the prognosis of GC patients and formulate personalized treatment plans.

## Introduction

Gastric cancer (GC) is the fifth most common cancer and the third most common cause of cancer death worldwide (1), and is one of the most common malignant tumors of the digestive system (2). However, GC at an early stage of discomfort symptoms are less or not obvious. Most GC patients have reached the stage of inoperable radical treatment at the time of diagnosis. Therefore, timely adjustment of clinical treatment strategy according to prognostic monitoring plays a crucial role in the treatment of GC (3). Tumor pathology (T), lymph node biopsy (N) and distant organ metastasis (M) are the main criteria to determine the prognosis of patients at present, but the prognosis of patients under the same TNM stage is also very different (4). In recent years, it has been observed that the prognosis of GC is not only related to the pathological stage, but also the tumor immune state may have an important influence on the prognosis of patients (5).

The immune microenvironment including anti-epidemic effector cells and molecules, plays an important role in the occurrence, development and clinical outcome of cancer. In recent years, tumor immunotherapy, as a novel treatment method, based on the human immune system, has demonstrated remarkable clinical effects by using immunoregulation to play an anti-tumor role. Immunotherapies such as immune checkpoint inhibitors and monoclonal antibodies can modulate the immune function of the body by changing the tumor microenvironment and producing an anti-tumor immune response (6). In GC, antibodies against PD-1 or PD-L1 can reverse the formation of tumor immunosuppressive microenvironment and exert anti-tumor effects (7). Immune cell infiltration is a major factor affecting the prognosis of GC. Foxp3-expressing regulatory T (Treg) cells into tumor tissue has been shown to be associated with poor prognosis in cancer patients (8). A variety of tumor immunotherapies related to natural killer cells have also entered the clinical trial stage (9). However, the abnormal changes in the gene expression profile related to immunity and the molecular mechanism of tumor immunity remain unclear (10). Continued detailed analysis of the immunological signatures of the tumor microenvironment will help to rapidly develop multiple novel immunotherapeutic strategies and identify potential biomarkers for clinical benefit (11).

Although many prognostic biomarkers have been discovered, such as carcinoembryonic antigen (CEA), carbohydrate antigen 199 (CA199), carbohydrate antigen 724 (CA724), which have been widely used in clinical practice (12). However, their effectiveness can be affected by various factors, and the prediction ability of a single indicator is insufficient (13). Instead, genetic markers provide better predictive performance, such genetic signatures are widely used for molecular diagnosis, individualized therapy, and accurate survival prediction (14), and multi-gene prognostic models can guide clinicians in choosing more appropriate treatments (15). Considering the significance of immunity, it is reasonable to conclude that IRGs has broad promise of GC prognostic evaluation, and that the polygene signatures generated by various algorithms will be better than single molecules in predicting prognosis. Numerous researchers have conducted research in this area. For example, Xie et al. (16) recently reported a 12 immue-related signature based on patients with breast cancer.

With the development of high-throughput sequencing technology, the combination of microarray data and bioinformatics has been widely used to identify a variety of cancer prognostic targets (17). In this study, based on immune-related genes that can reflect the infiltration of immune cells, a novel and reliable multi-gene risk scoring model was established to improve the prognosis of GC patients. Identification of core immune genes and pathways provides new therapeutic targets and generates new insights about GC progression.

## Materials and Methods

### Data Source

The RNA sequencing data and clinical data of all GC patients were downloaded from the Cancer Genome Atlas (TCGA) database (https://cancergenome.nih.gov) and Gene Expression Omnibus (GEO) database (http://www.ncbi.nlm.nih.gov/gds) including GSE84437, GSE57303. The immune gene data were obtained from the Immunology Database and Analysis Portal (ImmPort) (https://immport.niaid.nih.gov). The tumor-related transcription factor data were derived from the Cistrome (http://cistrome.org/) database. For TCGA and GEO data sets, the expression profiles were converted from probe level to the corresponding gene symbols based on each group of annotation files, and further standardized. By examining the data, only patients with complete clinical survival information were used for follow-up analysis.

### Identification of DEIRGs and Differentially Expressed Tumor-Related Transcription Factors

Differentially expressed genes (DEGs) in GC and normal tissues were screened from the gene matrix downloaded from the TCGA database using Package “limma” of R4.0.5 software (The screening criteria were FDR <0.05 and |log2FC| > 1). The intersection of IRGs and DEGs was used to obtain the DEIRGs in GC and normal tissues. Similarly, DETFs in GC and normal tissues were identified.

### Enrichment Analysis of DEIRGs and Protein Interaction Analysis

In order to annotate and analyze the biological functions of prognostic immune genes, we used the DAVID6.8 database (https://david.ncifcrf.gov) to perform Gene Ontology enrichment analysis (GO) and Kyoto Encyclopedia of Genes and Genomes (KEGG) Pathway analysis, the enrichment results with *P* < 0.05 were retained. Among them, KEGG utilizes its comprehensive database resources to analyze genetic information and make quantitative predictions on higher-level and more complex cell activities and biological behaviors. GO analysis selects three major categories: Biological Process (BP), Cell Component (CC) and Molecular Function (MF) to display the enrichment results. The protein interaction network was established by using STRING (http://string-db.org), and the interaction parameters were set as a composite score >0.9. Cytoscape software was used to reconstruct the network and cluster filtering was performed using the MCODE plug-in: Degree cut-off = 2, node score cut-off = 0.2, Max depth = 100, *k*-score = 2.

### Identification and Analysis of Prognostic Differentially Expressed IRGs

In the data set GSE84437, except for patients with incomplete clinical information and survival time <30 d, a total of 431 patients with GC were obtained. The samples were randomly divided into a training set (216 cases) and a test set (215 cases). In the training set, the PDEIRGs were evaluated by univariate Cox regression analysis. Meanwhile, by calculating the correlation coefficient between DETFs and PDEIRGs in GC, the regulatory network in GC was constructed. In addition, KEGG was used to explore the underlying immune molecular mechanisms and immune pathways.

### Establishment and Verification of a Prognostic Risk Scoring Model

In the training set, Lasso regression analysis was used to further analyze the PDEIRGs, so as to avoid over-fitting of the model. Based on the lambda.min method, the best possible PDEIRGs that may be used to establish the model were selected. According to the multivariate Cox regression analysis, the hazard ratio (HR) of each gene was calculated, and the final prognostic risk scoring model was established based on the linear combination of expression levels: Risk score (RS) = β1^*^expRNA1+ β2^*^expRNA2+…+βn^*^expRNAn, among them, β is the regression coefficient of the corresponding factor obtained by multivariate Cox regression analysis. ExpRNA is the expression of the corresponding RNA.

The sample RS was calculated according to the formula, and the patients with GC were divided into high-risk and low-risk groups based on the median value of the sample risk value. In order to further confirm and verify the prognostic value of the model, we used the R language “survival” package for Kaplan-Meier analysis and log-rank test in the training set to evaluate the survival time of the two groups of patients. Besides, we drew the ROC curve with the “survivalROC” package to verify the accuracy of the model. At the same time, the “pheatmap” package was adopted to draw the risk curve. In the same way, internal verification was carried out in the testing set and the overall set, while external verification was carried out in the GSE57303 and TCGA data sets. Then, based on the above model, we built a nomogram using “rms” package to better predict the prognosis of patients with GC.

### Correlation Analysis Between 9-Gene Signature and Immune Cell Infiltration

In order to figure out whether this model can reflect the status of the tumor immune microenvironment of patients with GC, we explore the correlation between 9-gene signature and the amount of immune cell infiltration through TIMER (https://cistrome.shinyapps.io/timer/) immune cell database and CIBERSORT. The Pearson correlation coefficient test was used to estimate the relationship between the expression of model genes and the content of different types of immune cells.

### Statistical Methods

All statistical analysis used R software 4.0.5 (Institute for Statistics and Mathematics, Vienna, Austria; https//www.r-project.org/). The following R software packages were used for further data analysis: limma, survival, survivalROC, pheatmap, rms etc. For all tests, *P* < 0.05 was considered statistically significant.

## Results

### Identification of DEIRGs and DETFs in GC

**Figure 1:**
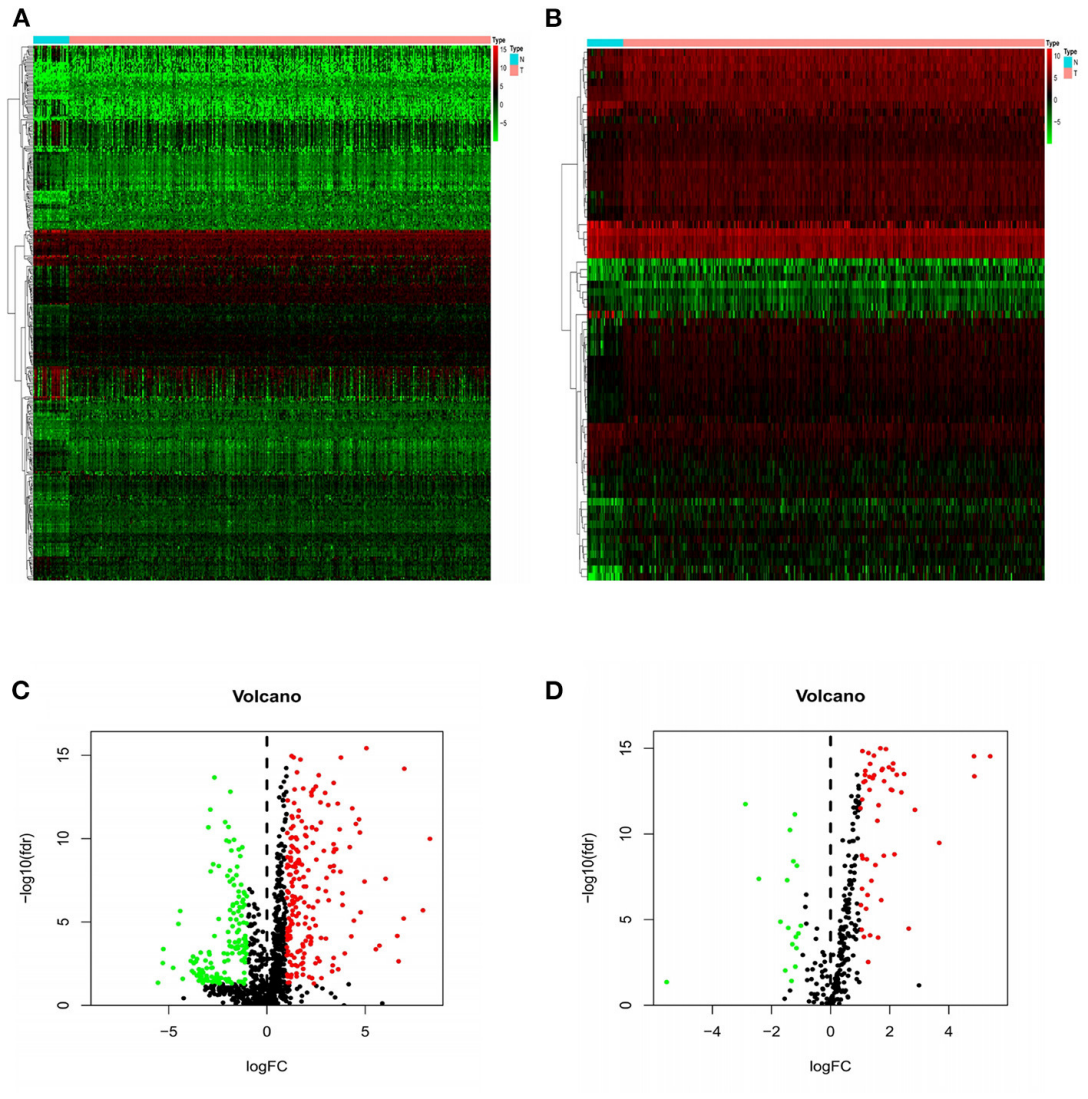
Heat maps and volcanic maps of IRGs and TFs expression. The differential expression heat maps of IRGs **(A)** and TFs **(B)**. The differential expression volcanic maps of IRGs **(C)** and TFs **(D)**. The heat map abscissa represents the sample: the blue area represents normal tissue and the red area represents tumor tissue; the ordinate represents the gene. On the volcano map, the green area represents the down-regulated differential genes and the red area represents the up-regulated genes.

The Wilcoxon rank sum test was used to analyze 375 GC and 32 normal gastric samples in TCGA GC data set. A total of 6,739 DEGs were screened by differential expression analysis (FDR <0.05, |log2FC| > 1). In addition, 2,483 IRGs were downloaded from the ImmPort database containing 17 categories, including tumor necrosis factor family members, transforming growth factor β family members, chemokines and cytokine receptors, interleukins, interferons, etc. In R language, 357 DEIRGs were obtained by intersection of IRGs and all DEGs, including 204 up-regulated DEIRGs and 153 down-regulated DEIRGs. The heat map and volcano map of DEIRGs were drawn (Figures 1A,C). In the same way, DETFs in GC tissues were analyzed. Compared with adjacent tissues, 71 DETFs were found in GC tissues, and the heat map and volcanic map of DETFs were plotted (Figures 1B,D).

### Functional Enrichment Analysis and Establishment of PPI Network of DEIRGs

**Figure 2:**
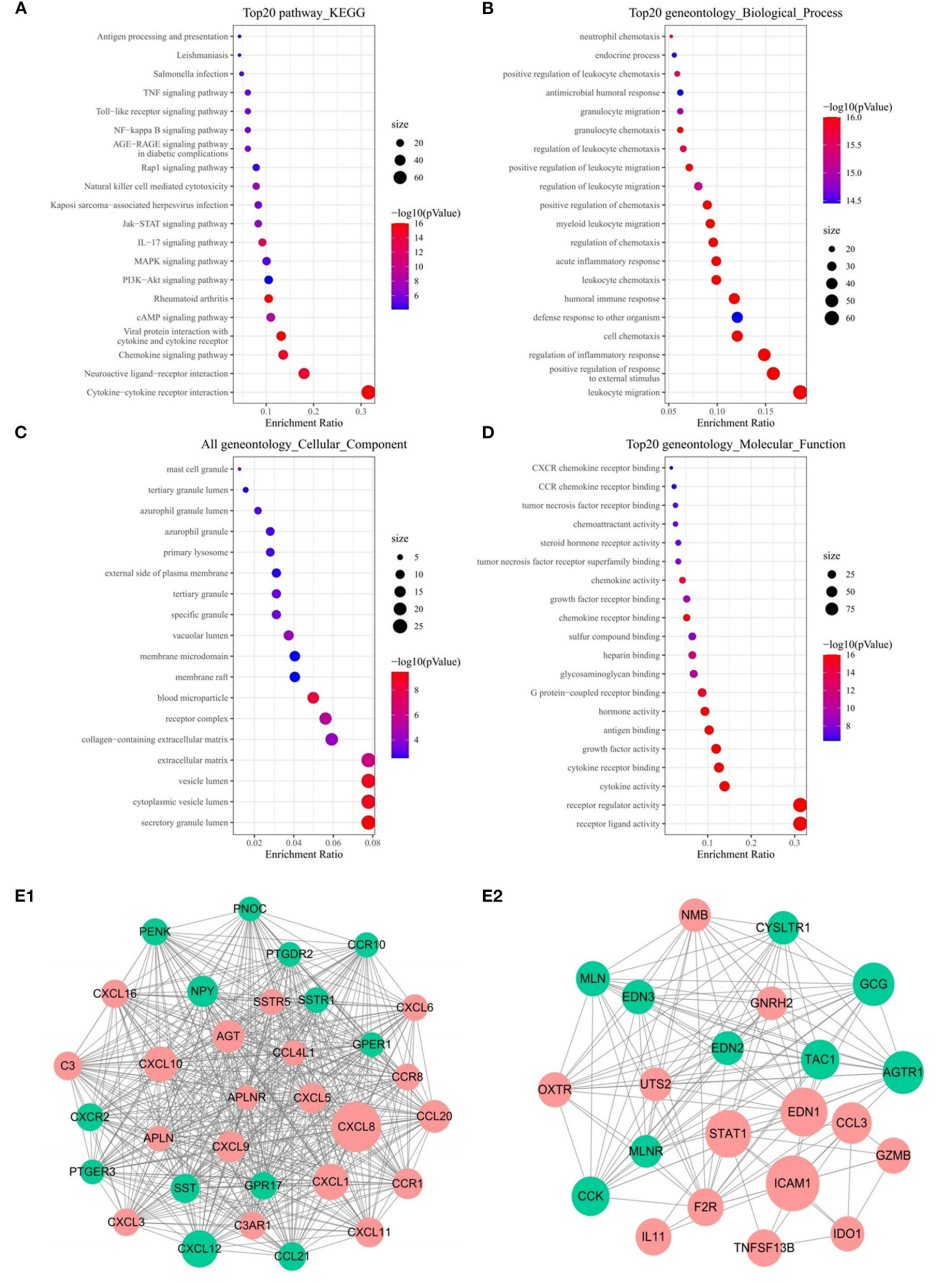
Functional enrichment analysis of 357 DEIRGs and significant modular analysis based on PPI network. Top 20 KEGG terms **(A)**. Top 20 GO terms of GO: BP **(B)**. Top 20 GO terms of GO: CC **(C)**. Top 20 GO terms of GO: MF **(D)**. PPI network of the APLNR module **(E1)**. PPI network of the STAT1 module **(E2)**. In A, B, C and D, terms are sorted by the number of genes enriched. In **(E1,E2)**, red stands for up-regulated and green stands for down-regulated genes. The size of the node represents the number of proteins that interact with the specified protein.

Functional analysis including KEGG and GO pathways was performed on these DEIRGs. KEGG pathway analysis revealed that these genes are mainly enriched in the cell cycle and vascular smooth muscle contraction process (Figure 2A). GO analysis showed that the biological processes of these genes are mainly involved in: muscle system processes, organelle fission, DNA-binding transcription activation activity, RNA polymerase II-specific binding to glycosaminoglycans, and the main cell components are: extracellular matrix and collagen extracellular matrix (Figures 2B–D).

PPI network was established based on 357 DEIRGs, and then a plug-in called MCODE of Cytoscape was used to identify the most significant collaborative regulation module. The score value of a node reflected the density of the node and the surrounding nodes. The top 2 significant modules were selected and are shown in Figures 2E1,E2, of which APLNR and STAT1 called seed genes that had the highest score in the modules. For the sake of convenience, we named these modules the APLNR and STAT1 modules, respectively. In the APLNR module, 496 edges involving 32 nodes were formed in the network, and CXCL8, CXCL12, CXCL1 CXCL10 were the remarkable nodes, as they had more connection with other genes of the module. The STAT1 module had 22 nodes with 127 degrees, and ICAM1, EDN1, STAT1, and GCG had higher degree values. Based on the online Kaplan-Meier Plotter database, it was found that high expression of APLNR was associated with poor prognosis of GC, while high expression of STAT1 was associated with good prognosis of GC (both *P* < 0.005) (Supplementary Figure 1).

### Identification of PDEIRGs and Establishment of a Regulatory Network

**Figure 3:**
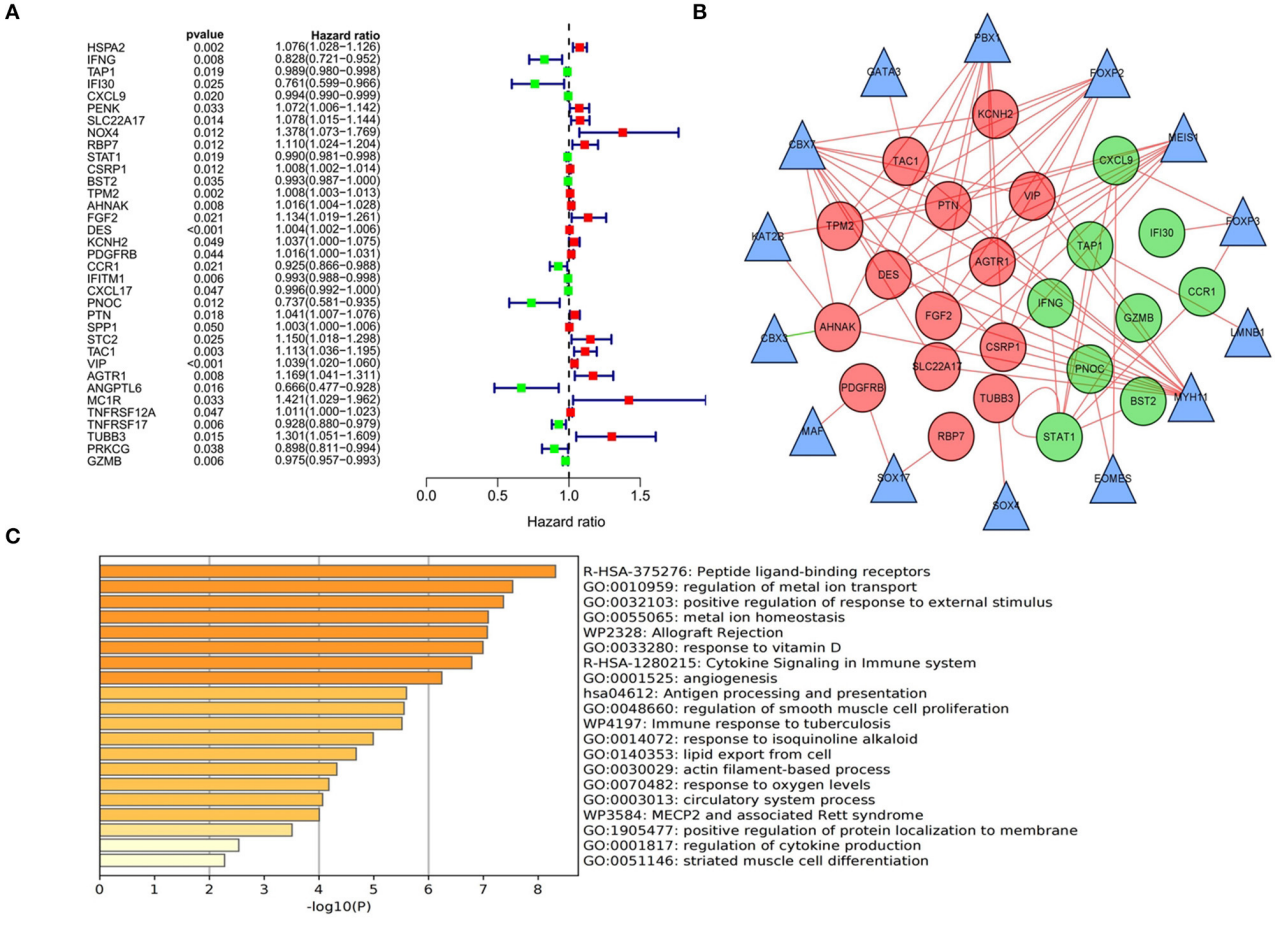
Identification, functional enrichment of PDEIRGs and construction of TF-based regulatory network in GC. **(A)** A forest diagram of PDEIRGs through univariate Cox analysis. The red mark indicates that HR value of the immune gene is >1 (high risk), and the green mark indicates that HR value of the immune gene is <1 (low risk). **(B)** TF-based regulatory network. The red and green dots represent PDEIRGs, the triangles represent DETFs that correlated with PDEIRGs in terms of their mRNA expression. **(C)** Functional analysis.

Due to the more sufficient number of patient samples in the GSE84437 data set, the expression of 357 DEIRGs identified in TCGA database was analyzed by univariate Cox analysis to identify PDEIRGs. The data showed that the expression of 35 DEIRGs was significantly related to OS in patients with GC (all *P* < 0.05) (Figure 3A). In order to further explore the role of PDEIRGs in the regulatory network, the relationship between PDEIRGs and DETFs was analyzed. The correlation between 71 DETFs and 35 PDEIRGs was detected (correlation coefficient > 0.4 and *P* < 0.001). The results showed that there was a significant correlation between 14 DETFs and 23 PDEIRGs (Supplementary Table 1). In addition, Cytoscape software was utilized to develop a TFs regulatory network to reveal direct correlations (Figure 3B). In order to explore the potential immune molecular mechanisms and immune pathways underlying the PDEIRGs, we conducted a functional analysis. It showed that they mainly focused on peptide ligand-binding receptors, positive regulation of response to external stimulus, allograft rejection, angiogenesis, etc. (Figure 3C).

### Establishment of a Prognostic Risk Scoring Model Based on IRGs

**Figure 4:**
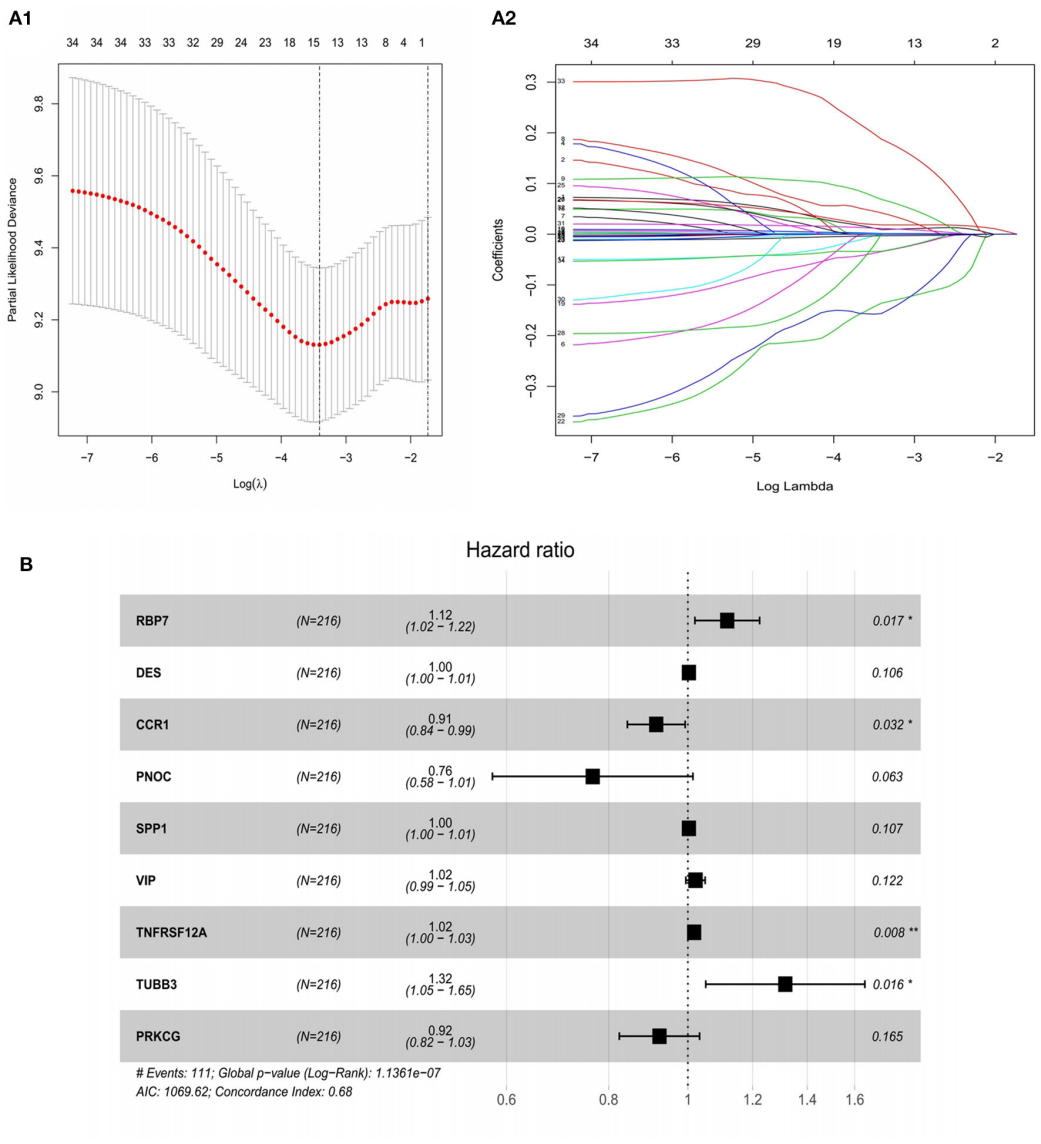
Construction of a risk assessment model by Lasso and Cox regression analysis in the training group. **(A1)** Selection of the optimal parameter (lambda) in the Lasso model for GC. **(A2)** Lasso coefficient profiles of genes in GC. A coefficient profile plot was generated against the log (lambda) sequence. **(B)** After optimization by Cox analysis, 9 mRNAs were selected to construct the risk assessment model as shown in the forest graph. ^**^P < 0.01; ^*^P < 0.05.

In order to identify the best model for predicting prognosis, we used Lasso regression analysis and multivariate Cox regression analysis to perform screening of variables on high-dimensional mRNA expression profile data. After 100 times of 10-fold cross-validation and optimization by Cox analysis (Figures 4A1,A2), the 9 genes (RBP7, DES, SPP1, VIP, TNFRSF12A, TUBB3, CCR1, PNOC, and PRKCG) obtained were determined as risk genes in the prognosis model as shown in the forest graph (Figure 4B). The high-risk genes were negatively correlated with the prognosis of patients, and the low-risk genes were positively correlated with the prognosis of patients. In the model, RBP7, DES, SPP1, VIP, TNFRSF12A, and TUBB3 were high-risk genes, and CCR1, PNOC, and PRKCG were low-risk genes. Extracting the coefficients of multivariate Cox analysis of mRNAs from the Lasso and Cox regression model: Risk score = (0.111005431) ^*^ RBP7 expression + (0.002868645) ^*^ DES expression + (−0.089069349) ^*^ CCR1 expression + (−0.26816701) ^*^ PNOC expression + (0.003158221) ^*^ SPP1 expression + (0.021454067) ^*^ VIP expression + (0.017371273) ^*^TNFRSF12A expression + (0.274538183) ^*^ TUBB3 expression + (−0.080069838)^*^PRKCG expression.

**Figure 5:**
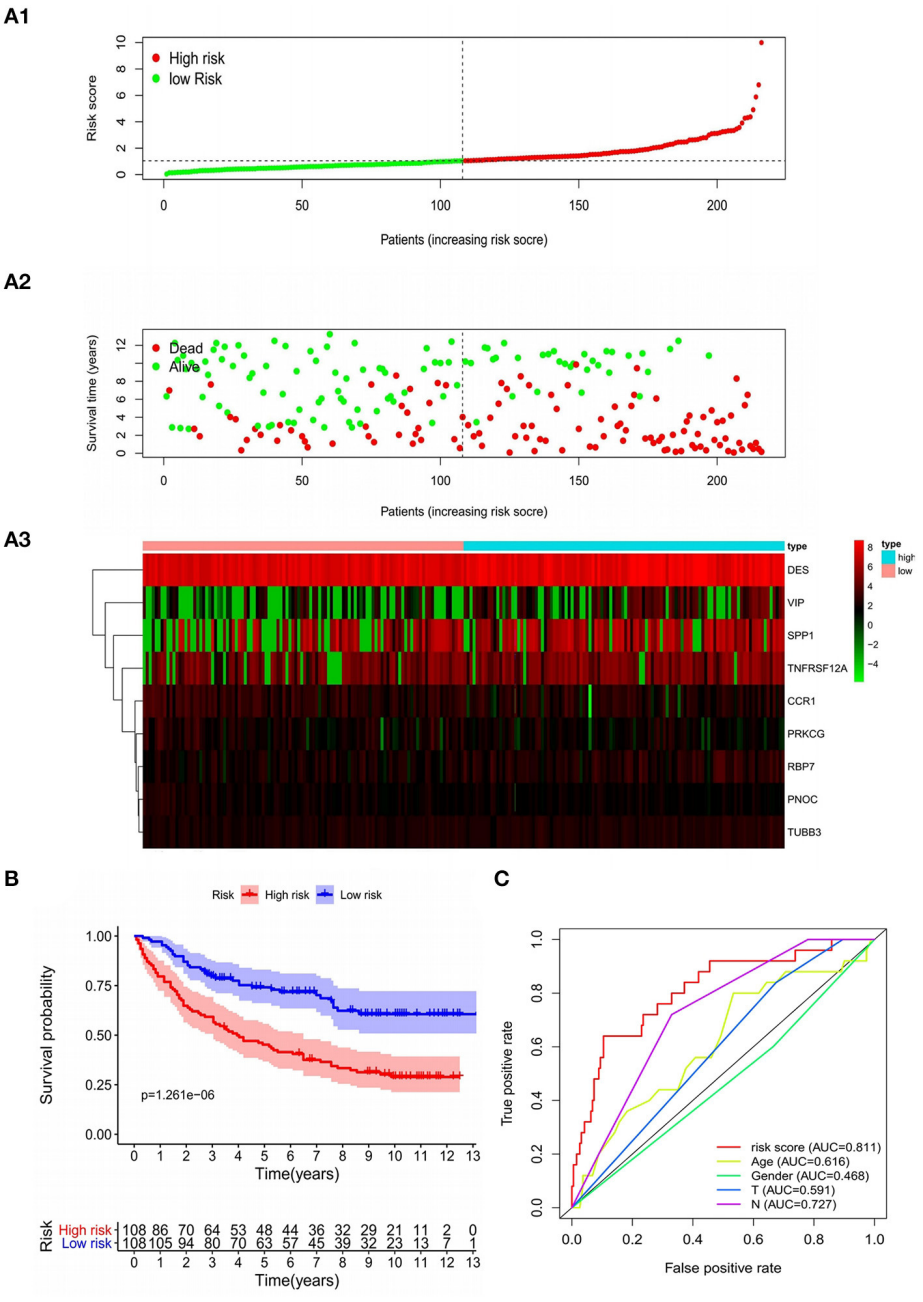
Characteristics of the prognostic model in the training group. **(A1,A2)** The distribution of risk score, patients' survival and status for GC. The black dotted line divided patients into high-risk group and low-risk group. **(A3)** Heat map of gene expression profiles in prognostic model for GC. **(B)** Kaplan-Meier survival analysis of patients stratified by the median risk score. **(C)** The ROC curve was applied to assess the predictive performance of the model compared to other clinical features.

In the training set, patients were classified according to the risk score and divided into high-risk group and low-risk group. The risk score distribution and survival status of high-risk and low-risk patients were shown in the Figures 5A1–A3. The survival time of GC patients decreased with the increase in risk score. Survival analysis was performed in high-risk and low-risk groups with R “survival” package. The Kaplan–Meier curve showed that the prognosis of patients in high-risk group was poorer. The 5-year OS of high-risk patients is 44.3%, while the 5-year OS of low-risk patients is 73.0%, the difference is statistically significant (*P* = 1.261e−06) (Figure 5B). In order to further verify the accuracy of the prognosis evaluation model and other clinical indicators, we used the R “survivalROC” package to draw multiple ROC curves. The results showed that the AUC of the model was 0.811 (Figure 5C), which was significantly higher than other clinical indicators, indicating that the risk scoring model had a satisfying predictive performance.

### Verification of Independent Risk Factors for GC

In addition, in the training set, we used the pathological characteristics in clinical data as the independent variable and patient survival time as a dependent variable for Cox univariate regression analysis. The results suggested that T stage, N stage and prognostic risk model were high-risk factors affecting the prognosis of GC. Taking the risk factors of GC as an independent variable and survival time as a dependent variable, the Cox multivariate regression analysis was conducted. The results indicated that the risk score of the risk scoring model was an independent risk factor affecting the prognosis of patients with GC (Table 1).

**Table 1:** Univariate and multivariate Cox regression analysis of the training set.

**Covariates**	**Univariate analysis**		**Multivariate analysis**	
	**HR(95% CI)**	***P***	**HR(95% CI)**	***P***
Age	1.016 (0.999–1.033)	0.062056	1.021 (1.003–1.038)	**0.02092**
Gender	1.129 (0.758–1.680)	0.550738	1.003 (0.657–1.531)	0.987295
T	1.597 (1.156–2.205)	**0.00447**	1.436 (1.025–2.011)	**0.03557**
N	1.669 (1.356–2.054)	**1.33E−06**	1.458 (1.168–1.818)	**0.00084**
riskScore	1.746 (1.537–1.983)	**1.01E**–**17**	1.674 (1.470–1.907)	**7.46E**–**15**

*Bold indicates that the result is meaningful*.

### Internal Validation of Prognostic Risk Scoring Model

**Figure 6:**
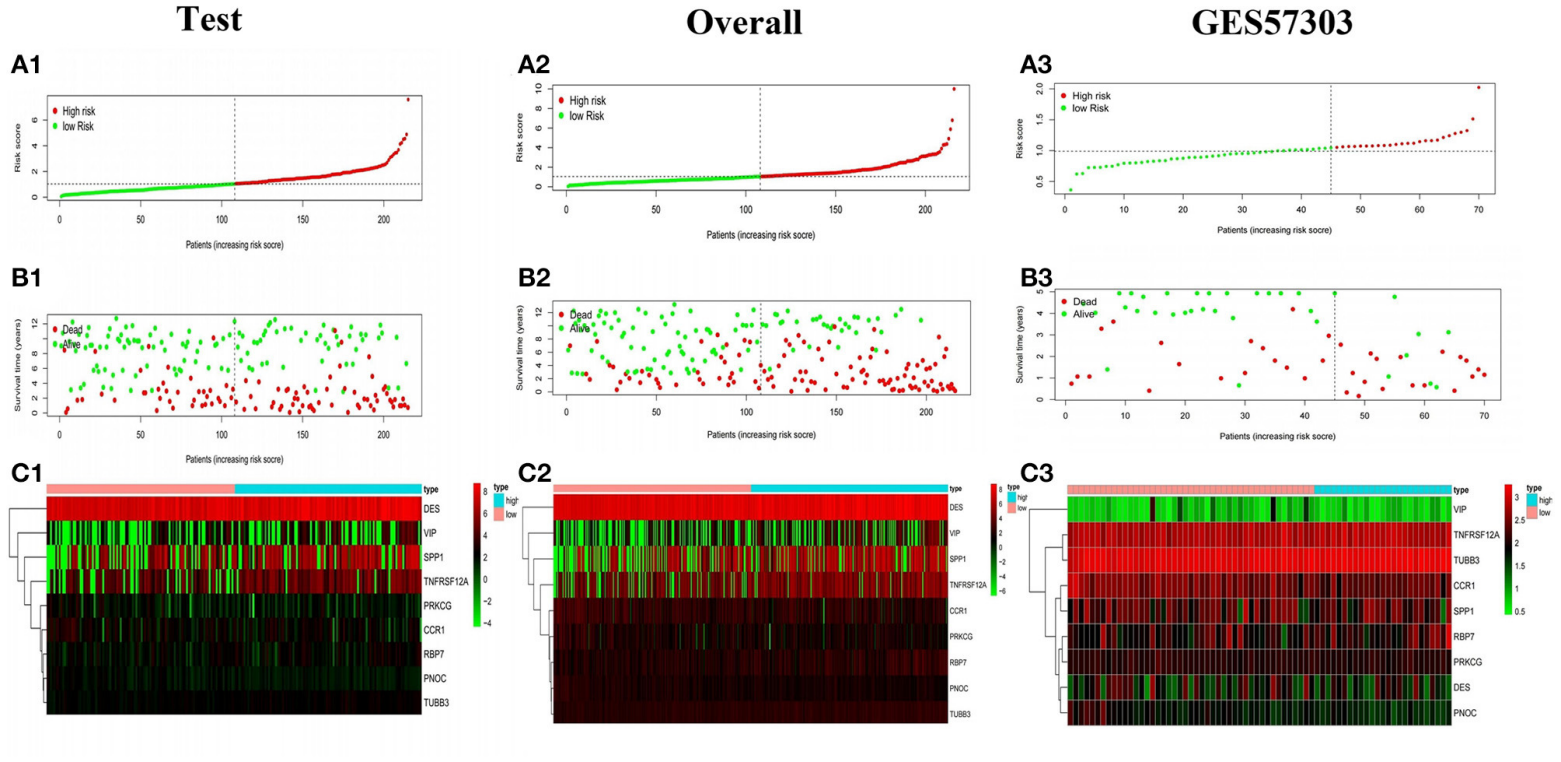
Survival status of gastric cancer patients with different risk scores. The distribution of risk score **(A1)**, survival time **(B1)**, heat map **(C1)** of the test set. **(A2,B2,C2, A3,B3,C3)** The distribution of risk score, survival time, heat map of the overall internal validation set and GSE57303 external validation set.

**Figure 7:**
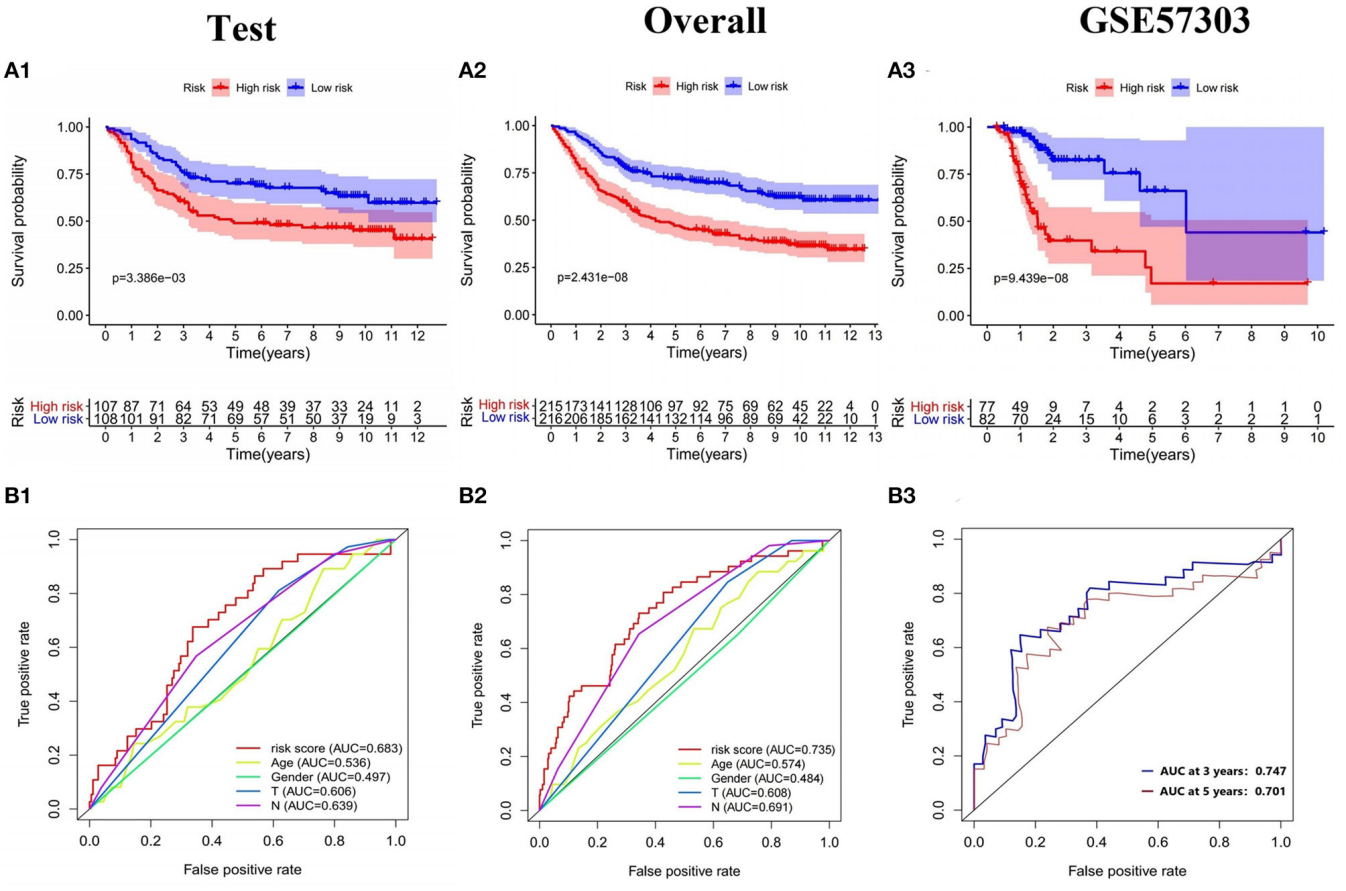
Validation of the prognostic model. **(A1–A3)** K-M survival analysis and **(B1–B3)** ROC curve of the test set, the overall internal validation set and GSE57303 external validation set.

The model was internally validated through the test set and the overall patients set. Firstly, 215 patient samples in the test set were classified according to the risk score of the model and divided into high-risk group and low-risk group. The risk score distribution and survival status of internal validation samples were shown in the Figures 6A1,B1,C1. The average survival time of cases was shorter and the number of deaths was larger in high-risk group, while the expression of 9 mRNAs in the high-risk group and the low-risk group was different. The Kaplan-Meier curve in the testing validation sample showed that the prognosis of patients in high-risk group was poorer (*P* = 3.386e−03) (Figure 7A1). The 5-year OS rate of high-risk patients was 49.0%, while that of low-risk patients was 70.0%. The area of the risk score of the internal validation sample under the AUC value of ROC was 0.683, which was significantly greater than other clinical indicators (Figure 7B1).

The training set and the testing set were combined into an overall patients set. As depicted in Figures 6A2,B2,C2, the survival status of high-risk patients is also not as good as that of low-risk patients, while the expression of 9 mRNAs in the high-risk group and the low-risk group was still different. As shown in the Kaplan-Meier survival curve (Figure 7A2), OS of patients in high-risk group was shorter than that in low-risk group (*P* < 0.001). The AUC of risk score was 0.735, which was significantly higher than other indicators (Figure 7B2).

### External Validation of Prognostic Risk Scoring Model

In addition, the model was externally validated in GSE57303 and TCGA GC. In GSE57303, 70 patient samples were classified according to the risk score of the model and divided into high-risk group and low-risk group. The risk score distribution and survival status of the external validation sample are consistent with the previous results (Figures 6A3,B3,C3). The Kaplan-Meier curve shows that the survival time of patients with low-risk score is significantly longer than that of patients with high-risk score (*P* = 9.439e−08) (Figure 7A3). The AUC values of the 3-, 5-year survival rate for patients were 0.747, 0.701, proving that the Cox model has a satisfying prognostic predictive ability (Figure 7B3). In TCGA GC containing 319 patient samples, the model still showed a good prognosis (Supplementary Figures 2A–C). Based on the clinical information of gastric cancer patients in TCGA database, including age, gender, stage, T, M, N, we constructed a prediction model of clinical predictive indicators based on Lasso regression analysis, and plotted the ROC curve of the model. The results showed that the AUC value was less than that of our 9-gene model. That is to say, the prediction performance of immune prediction model is superior to clinical prediction model (Supplementary Figure 4). The above all prove that the model had a reliable accuracy and the Cox model was preferable to other single indicators in predicting prognosis.

### Construction of the Nomogram

**Figure 8:**
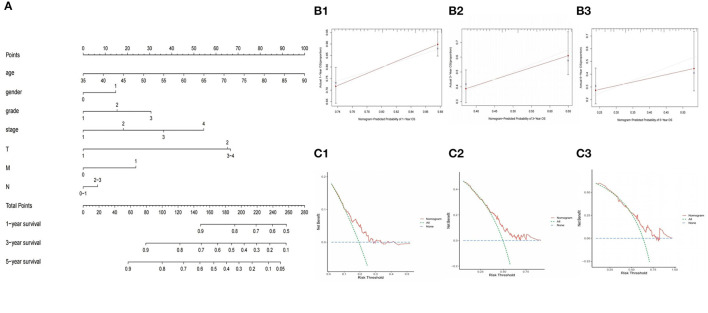
Construction and validation of nomogram to predict the probability of 1-year, 3-year, and 5-year survival rate in GC. **(A)** Nomogram of prognostic model. **(B1–B3)** Verification of nomogram capability in the TCGA database. **(C1–C3)** Verification of DCA curves in the TCGA database.

In order to provide clinicians with quantitative methods to predict the prognosis of GC patients, we established a nomogram using rms package based on TCGA-GC, which combined with the various predictors of the prognosis of GC patients, patient age and risk score (Figure 8A). Further, The calibration curves and DCA curves of 1, 3, and 5 years showed that the 1-, 3-, and 5-year survival rate of GC patients predicted by the nomogram coincides well with the actual survival rate, which indicated that the nomogram we have established can better predict the survival rate of GC patients (Figures 8B1–B3,C1–C3). In addition, the nomogram constructed based on GSE84437 has also been well-verified by the calibration curve (Supplementary Figures 3A,B).

### Correlation Analysis Between Model Genes and Pathological Characteristics

**Figure 9:**
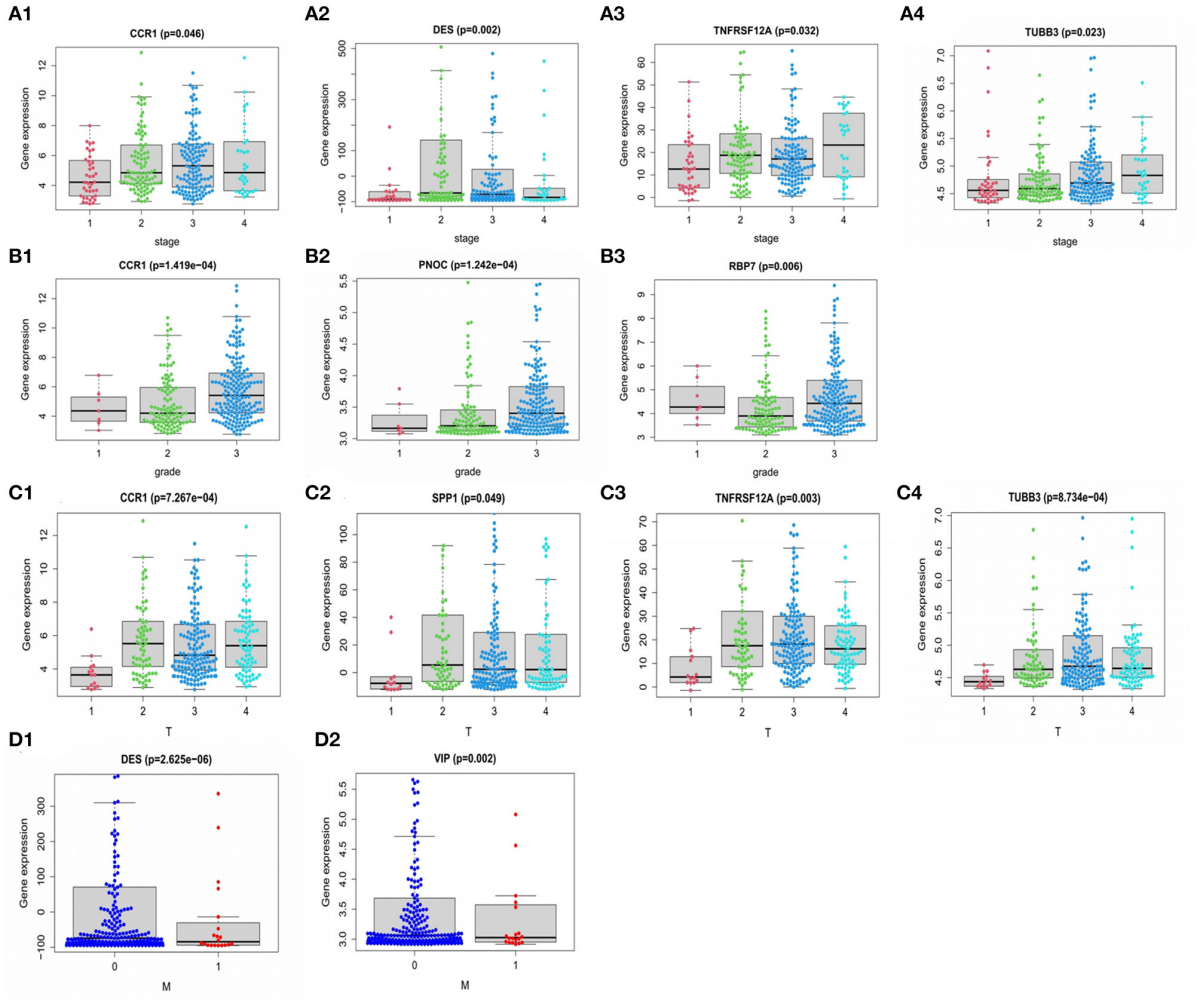
Relationship of PDEIRGs with patients' clinical characteristics of GC. **(A1–A4)** Immune gene CCR1, DES, TNFRSF12A, TUBB3 had significant clinical correlation with stage. **(B1–B3)** CCR1, PNOC, RBP7 had significant clinical correlation with grade. **(C1–C4)** CCR1, SPP1, TNFRSF12A, TUBB3 had significant clinical correlation with T stage. **(D1,D2)** DES, VIP had a significant clinical correlation with the M stage.

**Figure 10:**
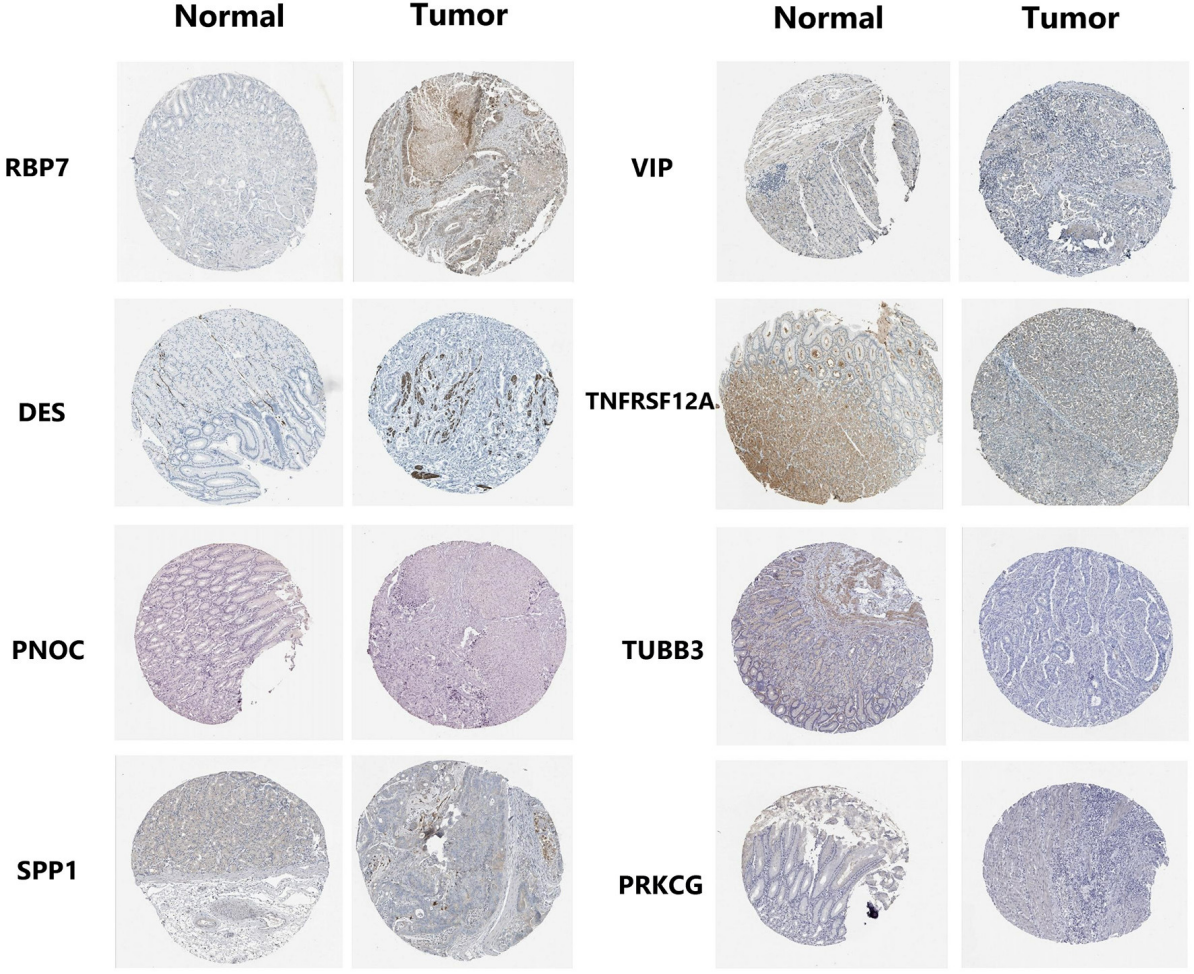
Expression profiles of 8 genes in normal stomach tissues and gastric adenocarcinoma tissues. Immunohistochemical results were obtained from HPA database.

GO and KEGG pathway analysis were employed to identify the biological function of the model genes (Table 2). The R language “Beeswarm” package was used to further study the relationship between gene distribution and clinical parameter stratified. It was found that the expressions of RBP7, CCR1, and PNOC were positively correlated with clinical grade. DES, CCR1, VIP, TNFRSF12A, and TUBB3 were positively correlated with the clinical stage. CCR1, SPP1, TNFRSF12A, and TUBB3 were positively correlated with T stage. DES and VIP were positively correlated with N stage (all *P* < 0.05) (Figure 9). In order to study the protein expression of 9 genes in normal tissues and gastric cancer tissues, we searched the Human Protein Atlas (HPA) database and obtained the immunohistochemical results of 8 genes (Figure 10). The results of immunohistochemistry showed that the protein expression levels of RBP7, DES, SPP1, VIP, and PRKCG in gastric cancer tissues were higher than those in normal tissues, while PNOC, TNFRSF12A, and TUBB3 proteins are less expressed in gastric cancer tissues than normal tissues. However, the immunohistochemical results of CCR1 were not found in the database.

**Table 2:** Enrichment analysis of genes in the model.

**Term ID**	**Term description**	**Strength**	**False discovery rate**	**Matching proteins in the network**
GO:0005184	Neuropeptide hormone activity	2.18	0.0082	PNOC, VIP
GO:0005200	Structural constituent of cytoskeleton	1.61	0.0461	TUBB3, DES
GO:0048018	Receptor ligand activity	1.15	0.0461	PNOC, VIP, SPP1
hsa04540	Gap junction	1.7	0.0461	PRKCG, TUBB3
HSA-1280215	Cytokine Signaling in Immune system	1.12	0.0472	CCR1, TNFRSF12A

### Correlation Analysis Between 9-Gene Model and Immune Cell Infiltration

**Figure 11:**
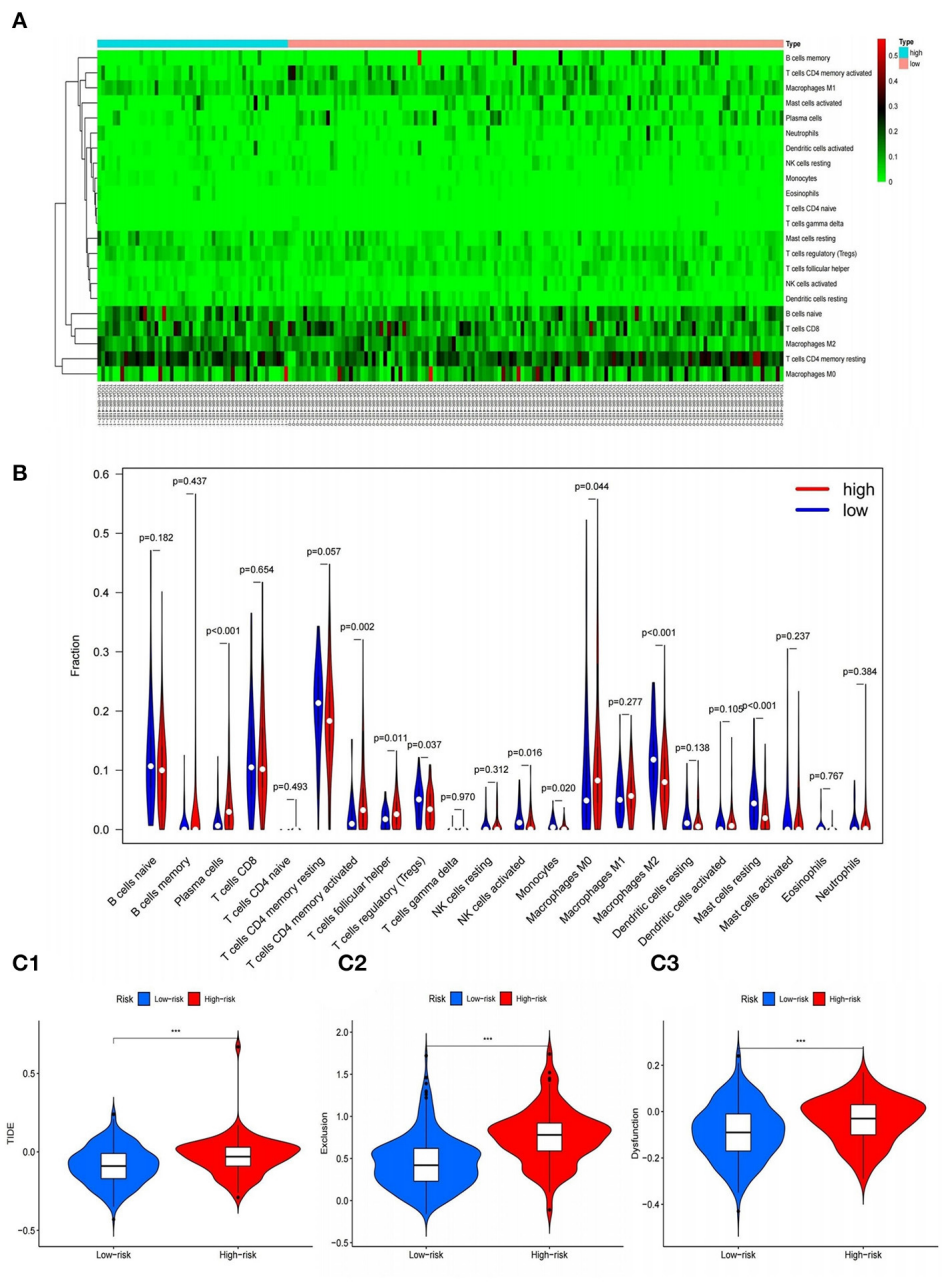
Correlation of gene model with immune cell infiltration and immunotherapy. **(A,B)** Heat and violin plots of differences in immune cell infiltration in high and low risk groups. **(C1–C3)** Violin plots of differences in TIDE between high and low risk groups. ^***^P < 0.001.

In order to further study the relationship between the 9-gene signature and immune cells, the gene expression data of the samples in the TCGA cohort and the gene expression profiles of 22 kinds of immune cells were analyzed by CIBERSORT software, and the contents of various immune cells in the samples were estimated. According to the risk value calculated by the model, patients are divided into high and low risk groups, we found that the risk score calculated based on 9-gene signature was found to be significantly correlated with the infiltration of 9 types of immune cells, including Plasma cells, T cells regulatory (Tregs), Macrophages M0, etc. (Figures 11A,B).

The correlation between RBP7, DES, CCR1, PNOC, SPP1, VIP, TNFRSF12A, TUBB3, PRKCG genes and the amount of immune cell infiltration was explored by TIMER. Spearman correlation analysis was used for correlation analysis. As shown in the table, the expressions of CCR1, PNOC, and VIP genes were significantly correlated with tumor purity, B cell infiltration, CD8+T cell infiltration, CD4+T cell infiltration, macrophage infiltration, neutrophil infiltration and dendritic cell infiltration, and the differences were statistically significant. There was a significant correlation between DES gene expression and tumor purity, B cell infiltration, CD8+T cell infiltration, CD4+T cell infiltration, macrophage infiltration, and dendritic cell infiltration, and the differences were all statistical significance. The expression of other genes was significantly related to tumor purity and the infiltration of one or more immune cells, and the differences were all statistically significant (all *P* < 0.05) (Supplementary Table 2).

### The Effect of Immunotherapy in High- and Low-Risk Groups

In addition, in order to explore the effect of immunotherapy in high- and low-risk groups, the tumor immune dysfunction and rejection (TIDE) calculation framework was used to simulate the two main mechanisms of tumor immune evasion and provide predictive results for immunotherapy. Elevated TIDE indicates that the patient has inhibitory cells that inhibit T cell infiltration and does not respond to immunotherapy. We found that the score of the high-risk group was significantly higher than that of the low-risk group, that is, there were more obvious immune dysfunction and immune rejection, indicating that the immunotherapy effect of patients in the high-risk group were often poor (Figures 11C1–C3).

## Discussion

GC is a type of highly heterogeneous malignant tumor, with a rising incidence worldwide, poor prognosis and high mortality (18). There is evidence that commonly used clinicopathological parameters (such as TNM stage, age, sex, viral infection, and serum CEA levels) are insufficient to accurately predict patient outcomes (19). Therefore, it is necessary to identify sensitive and stable GC prognostic biomarkers to improve treatment. At present, models based on genetic signatures have been reported in a variety of cancers, such as ovarian cancer, lung adenocarcinoma, and soft tissue sarcoma (20–22). Immunotherapy involves changes in genes, metabolism, inflammation and tumor microenvironment, and the mechanisms are complex. Considering that cell atypia and even canceration often occur in a microenvironment of dense inflammatory cells, the study of tumor genomics and changes in the tissue microenvironment is a new challenge (23).

Based on TCGA database, this study utilized R language to screen out the DEIRGs in GC tissues. Combined with detailed clinical data in GSE84437, Lasso and Cox analysis was applied to screen genes closely related to the prognosis of patients in 358 DEIRGs. In the end, 9 mRNAs were selected to form a prognostic model. Survival analysis, ROC curve and risk curve were used in the training set, validation set and external validation set to verify the accurate prediction performance of the model. In order to independently predict patient survival, the model was combined with other clinical variables to construct a nomogram for better clinical application. At the same time, we performed gene expression and clinical correlation analysis on the model genes discovered in the study, and found a number of genes closely related to the patient's clinical status, providing a basis for finding reliable genetic biological targets.

In tumor cells, an inactivated, pleiotropic transcription factor simultaneously coordinates the expression of a large family of responding genes, causing them to act together to produce the phenotype of the cancer cell (24). In our work, there is a statistically significant correlation between multiple DETFs and PDEIRGs. Transcription factors play a direct role in regulating immune-related genes. Among these TFs, FOX family and Chromobox family were found to be closely related to the occurrence and development of GC. Experiments have shown that Treg-like immunosuppressive effects can occur after inducing initial T cells to express FOXP3 *in vitro* or *in vivo*, indicating that FOXP3 is a key factor in controlling the expression of immunosuppressive molecules (25). High expression of CBX7 can enhance the clonal proliferation, migration and invasion of GC cells, promote the self-renewal of GC stem-like cells, and reduce the sensitivity of GC cells to chemotherapy drugs (26). It can be deduced that the targeted therapy of TFs will become an important direction of tumor research in the future.

Furthermore, the results of functional annotation showed that the genes in the model played key roles in receptor ligand activity, gap junction, and cytokine signal transduction. It is worth noting that mediated GJIC is one of the most common way of communication between cells (27), in regulating cell growth, differentiation and apoptosis plays an important role, inhibition of GJIC function or defects can make the former around tumor cells or cells lose normal cell growth regulation and autonomy, and then develop into tumor cells, It is an important mechanism of promoting carcinogenesis stage (28, 29). The research on the mechanism of the influence of model genes on gap junction can be regarded as our subsequent research direction. In addition, immune cell infiltration plays an important role in tumor development. Previous studies have found that the accumulation of tumor-associated macrophages is associated with poor clinical prognosis (30). Dendritic cells play a critical role in the initial activation of tumor immunity (31). The expression of multiple genes in the model was significantly correlated with the infiltration of various immune cells and tumor purity, which also indicated that our model could effectively predict the prognosis of GC patients.

According to previous studies, the genes identified in our work regarded as independent prognostic factors for GC were often found to be involved in tumor occurrence and development. CCR1 is a member of the β-chemokine receptor family. Chemokines are an important component of the tumor microenvironment. CC chemokines produced by tumor cells and tumor-related cells can affect the proliferation, migration and invasion of cancer cells (32, 33). Interestingly, this study found that the high expression of CCR1 was significantly related to the good prognosis of GC patients, whether CCR1 can participate in the GC process through chemokine antagonism is our next step. The protein of TUBB3 is mainly expressed in neurons and may be involved in neurogenesis and axon guidance and maintenance (34). Cao et al. (35) found that the expression of TUBB3 is significantly correlated with the clinicopathologic characteristics of gastric cancer patients, such as age, sex and family history. It may be a potential biomarker for prognosis and chemotherapy guidance. TNFRSF12A is the receptor of TNFSF12/TWEAK, which can promote angiogenesis and endothelial cell proliferation, and regulate cell adhesion to matrix proteins. Wu et al. (36) found that the decrease of TNFRSF12A expression in thyroid cancer indicates a poor prognosis. RBP7 encoded proteins are members of the cellular retinol binding protein (CRBP) family, and their members are necessary for vitamin A stability and metabolism (37). Previous studies have shown that RBP4, as a member of the same family, its receptor is a strong transformation medium and drives malignant transformation in human breast and colon cancer cells (38). Elmasry M's study (39) showed that RBP7 is also a clinical prognostic biomarker and is associated with tumor invasion and EMT of colon cancer. Moreover, in our work, we found that RBP7 is a high-risk factor for GC, so figuring out how RBP7 drives these malignant features and how it affects the transcriptome of gastrointestinal tumor cells is our next research direction.

In addition to these genes, several other genes in the study also have intriguing biological implications. VIP is a neuropeptide belonging to the family of G protein-coupled receptors, which can activate cAMP-response element binding protein by regulating the cAMP/PKA signaling pathway (40). It involves in gastric smooth muscle relaxation, vasodilation and gastric juice secretion. Zygulska et al. (41) found that overexpression of vasoactive intestinal peptide (VIP) receptors may occur in human surface gastrointestinal malignant tumors; Balkwill et al. (42) found that neurotransmitter secretion is increased in the inflammatory response caused by cancer. Based on these, it is inferred that the increased expression of VIP and its receptor may be related to cancer-related inflammation. SPP1 is primarily located in the extracellular matrix, mainly involved in the adhesion of cell matrix, osteogenization, anticellulation apoptosis, cell attachment, immunocyte chemokine, and other biological processes. According to reports, SPP1 can promote tumor cell survival, regulate tumor-related angiogenesis and inflammatory responses (43), closely related to lung cancer, ovarian cancer, colon cancer, head and neck squamous cancer and adverse prognosis closely related (44, 45). There has been no previous report on SPP1 and GC, but this study, based on bioinformatics, first found that SPP1 is a risk factor for GC prognosis, and its mechanism of tumor action needs further study and demonstration. Moreover, it is worth noting that STAT1 and APLNR are the seed genes in the module, and their role in tumors has been confirmed, which is consistent with the results of this study. It has been suggested that STAT1 serves as a tumor suppressor by promoting the expression of p21waf, caspase 3 and caspase 7 to activate pro-apoptotic pathways (46). APLNR is the receptor for Apelin. In various cancers, Apelin expression increases, and the Apelin/APLNR axis play an essential role in tumor development by enhancing angiogenesis, metastasis, cell proliferation, and cancer stem cell development and drug resistance (47).

Based on a search of previous literature, a prognostic model based on gene signatures has been established in a variety of cancers and its feasibility has been verified. However, few studies have integrated multiple data sets in GC to explore immune gene regulatory networks and establish reliable prognostic models, as well as in-depth analysis of its molecular mechanism. This study innovatively screened the immune genes with prognostic significance in GC, and combined with reliable statistical methods to construct a prognostic risk model based on 9 immune-related genes. Multiple data sets in different platforms proved that the model relative to other clinical staging method had more excellent performance and reliable prediction. In addition, we found that the model gene was not only related to GC stage and grade, but also reflected the immune microenvironment of GC, providing insights for immunotherapy. The traditional TNM gastric cancer staging system cannot be used to accurately predict whether patients will benefit from adjuvant chemotherapy. The establishment of this model completes the existing staging system, and can guide accurate prognosis judgments and treatment options.

However, this study also has some limitations. First, the risk scoring model needs to be further validated in multi-center clinical trials and prospective studies. Secondly, the function of 9 immune genes still needs to be further analyzed. In various studies on GC, the prognostic risk model of GC described in this article has not been reported previously. This study used bioinformatics methods to establish a prognostic model consisting of RBP7, DES, CCR1, PNOC, SPP1, VIP, TNFRSF12A, TUBB3, and PRKCG, and proved its prognostic value in GC. The nomogram established in combination with the risk score can clinically predict the OS of patients with GC after resection. It is worth noting that this study provides a new immunological perspective and a new basis for immunotherapy of GC. This model may be a new and accurate prediction tool for evaluating whether GC patients can benefit from immunotherapy.

## Conclusions

In summary, based on the TCGA and GEO data set, we have identified DEIRGs expressed in GC tissues. We established a reliable multi-gene joint prediction model, which is beneficial to improve the prognosis of patients. Moreover, this study found several prognostic markers worth exploring: APLNR is a risk factor for GC, so blocking the Apelin/APLNR axis to inhibit the development of gastric cancer is a promising new therapy. In addition, CCR1 and STAT1 are protective factors for gastric cancer. The enhancement of the pro-apoptotic pathway of STAT1 has the potential to inhibit GC. Whether CCR1 can participate in the GC process through chemokine antagonism is our next step.

## Data Availability Statement

The datasets presented in this study can be found in online repositories. The names of the repository/repositories and accession number(s) can be found in the article/Supplementary Material.

## Author Contributions

ML designed, analyzed the data, and wrote the manuscript. WC helped to prepare the dataset and participated in the discussion. YC and ZZ helped to search for some relevant papers for this research. BH analyzed the data and generated the figures and tables. BC and GC guided the research process. All authors read and approved the final manuscript.

## Funding

This work was supported by the National Natural Science Foundation of China (81602425), the Anhui Quality Engineering Project (2020jyxm0898 and 2020jyxm0910), the Anhui Health Soft Science Research Project (2020WR01003), and the Key Research and Development Program of Anhui Province (201904a07020045).

## Conflict of Interest

The authors declare that the research was conducted in the absence of any commercial or financial relationships that could be construed as a potential conflict of interest.

## Publisher's Note

All claims expressed in this article are solely those of the authors and do not necessarily represent those of their affiliated organizations, or those of the publisher, the editors and the reviewers. Any product that may be evaluated in this article, or claim that may be made by its manufacturer, is not guaranteed or endorsed by the publisher.

## Supplementary Material

Supplementary Figure 1 Kaplan-Meier analysis of the impact of the 2 seed genes on the prognosis of GC patients.

Supplementary Figure 2 Validation of the prognostic model in TCGA GC set.

Supplementary Figure 3 Construction and validation of nomogram in GEO database.

Supplementary Figure 4 ROC curve of predictive model based on clinical predictive indicators.

Supplementary Figure 5 Boxplot of differential expression of CCR1 in gastric cancer and normal tissues.

Supplementary Table 1 Correlation between TFs and immuneGenes.

Supplementary Table 2 Analysis of the relationship between PDEIRGs and immune cell infiltration.

Additional File 6 Differentially expressed immune-related genes of GC.

Additional File 7 Clinical information of patients in TCGA cohort.

Additional File 8 Clinical information of patients in GSE57303 cohort.

Additional File 9 Clinical information of patients in GSE84433 cohort.

Additional File 10 Correlation and regulatory relationship between TF and genes in the prognostic model.
