# Supplementary figures and images for: Establishment and Analysis of an Individualized Immune-Related Gene Signature for the Prognosis of Gastric Cancer
Source: Front Surg. 2022 Jan 31;9:829237. doi: 10.3389/fsurg.2022.829237 (PMC8841693; doi:10.3389/fsurg.2022.829237)

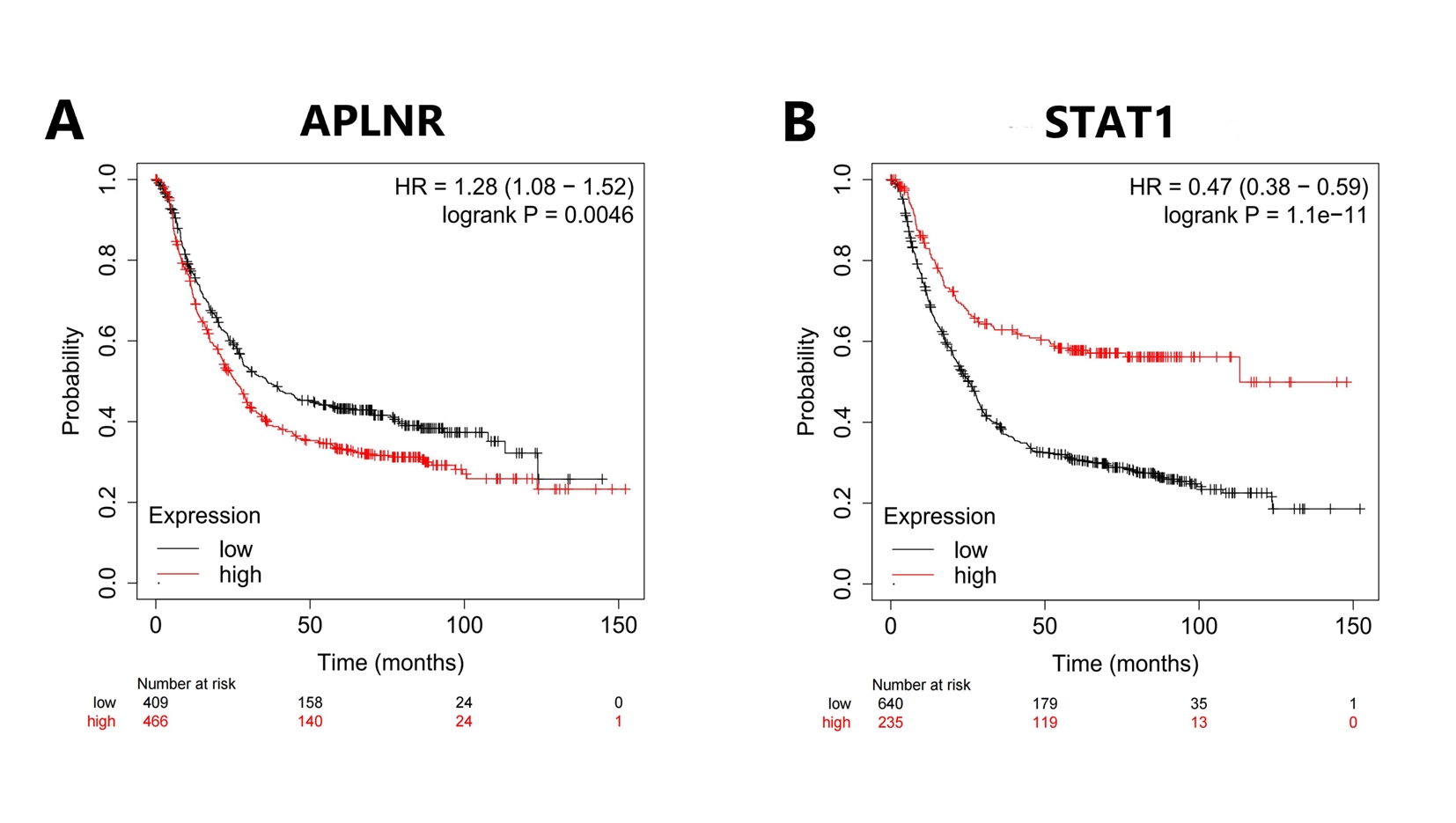

Supplement: Supplementary Figure 1 — Kaplan-Meier analysis of the impact of the 2 seed genes on the prognosis of GC patients. [file Image_1.JPEG]

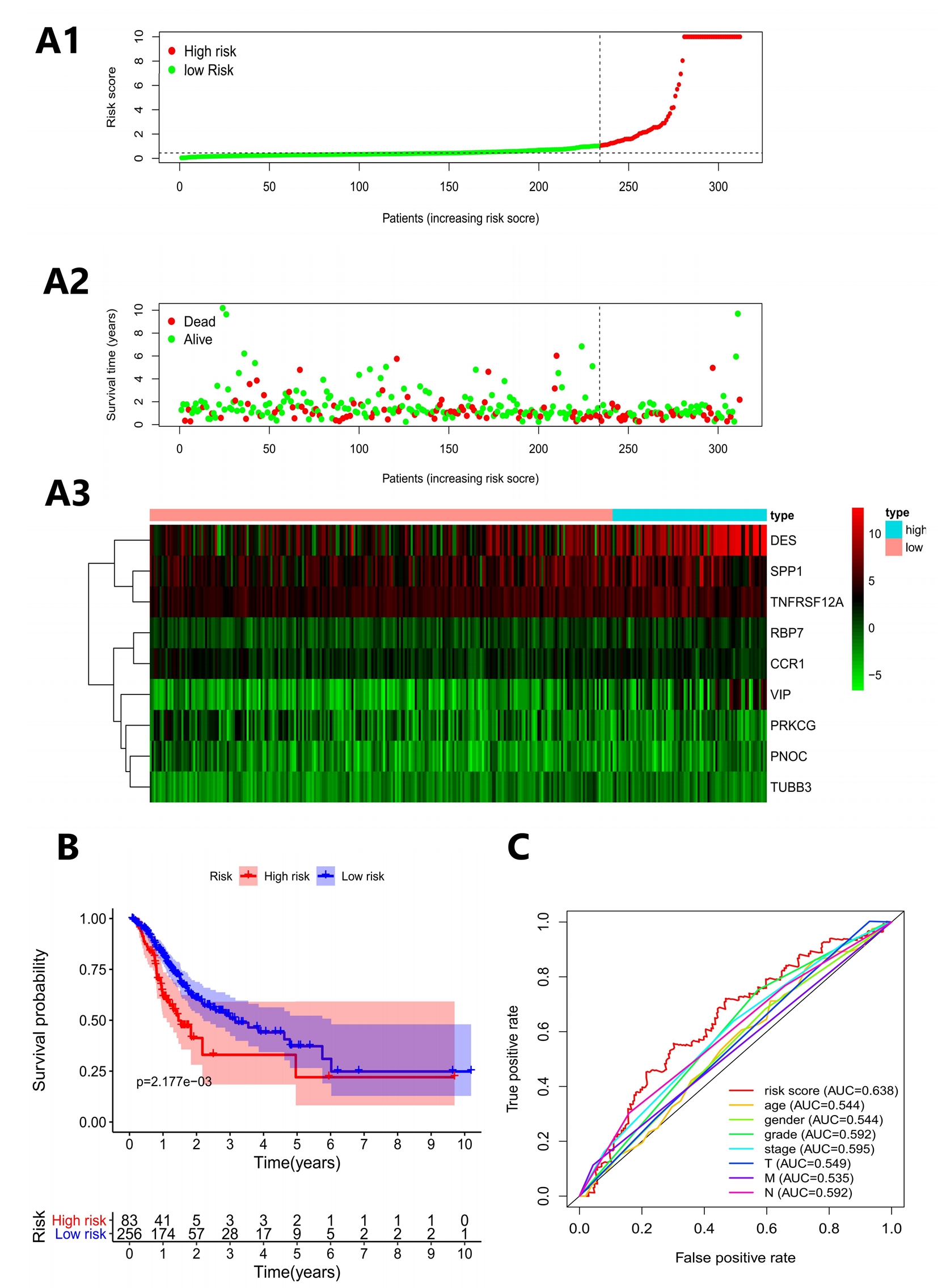

Supplement: Supplementary Figure 2 — Validation of the prognostic model in TCGA GC set. [file Image_2.JPEG]

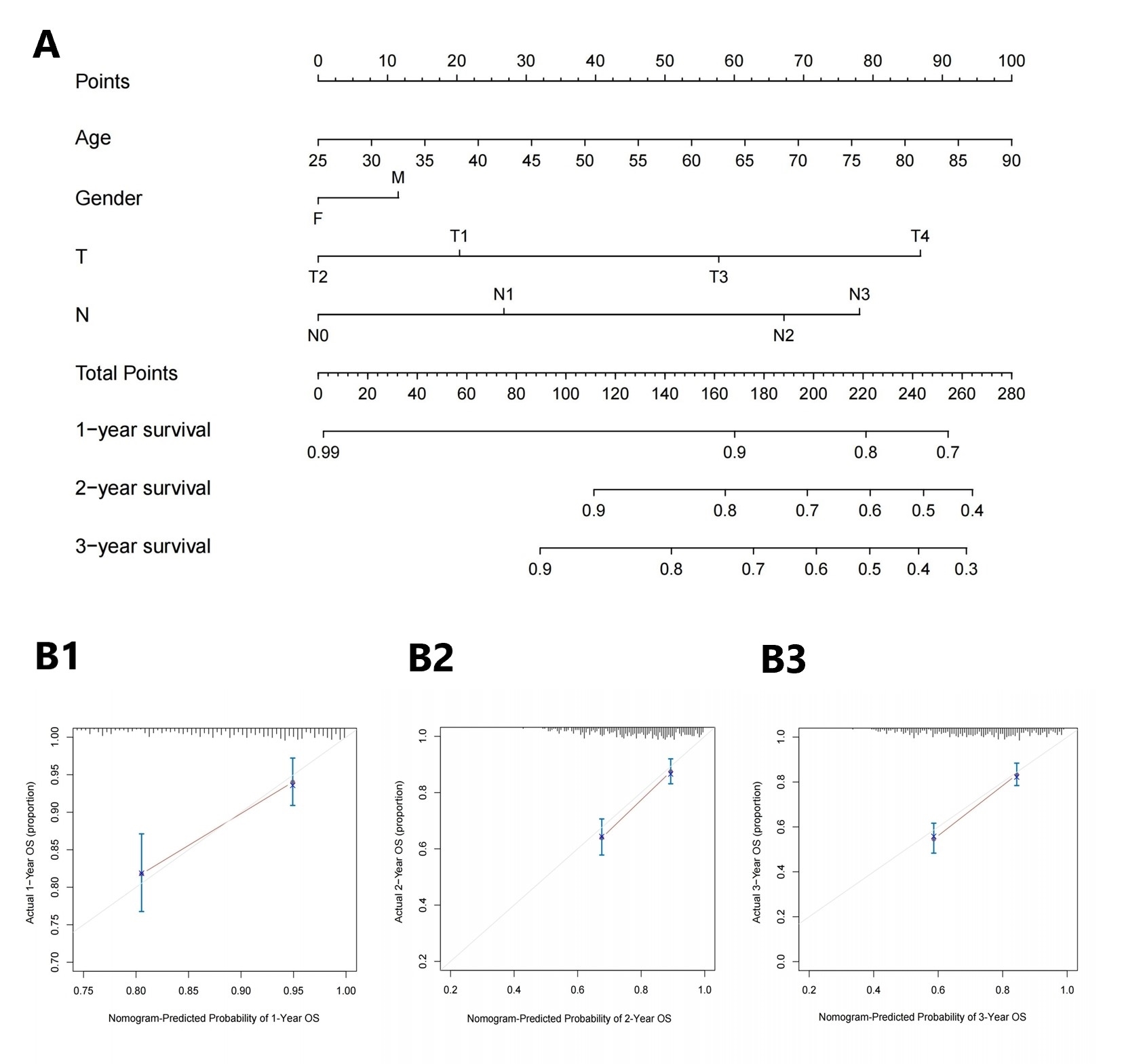

Supplement: Supplementary Figure 3 — Construction and validation of nomogram in GEO database. [file Image_3.JPEG]

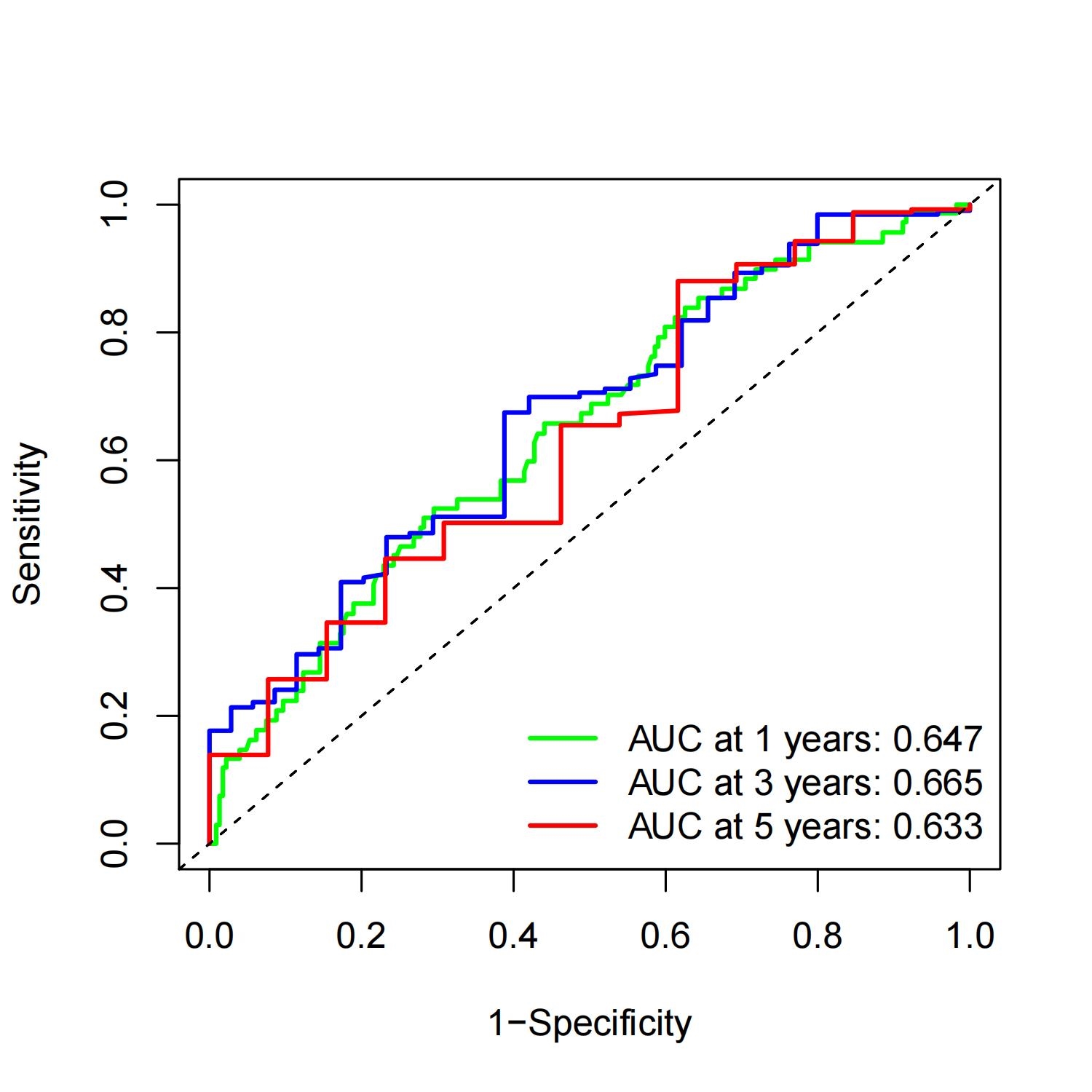

Supplement: Supplementary Figure 4 — ROC curve of predictive model based on clinical predictive indicators. [file Image_4.JPEG]

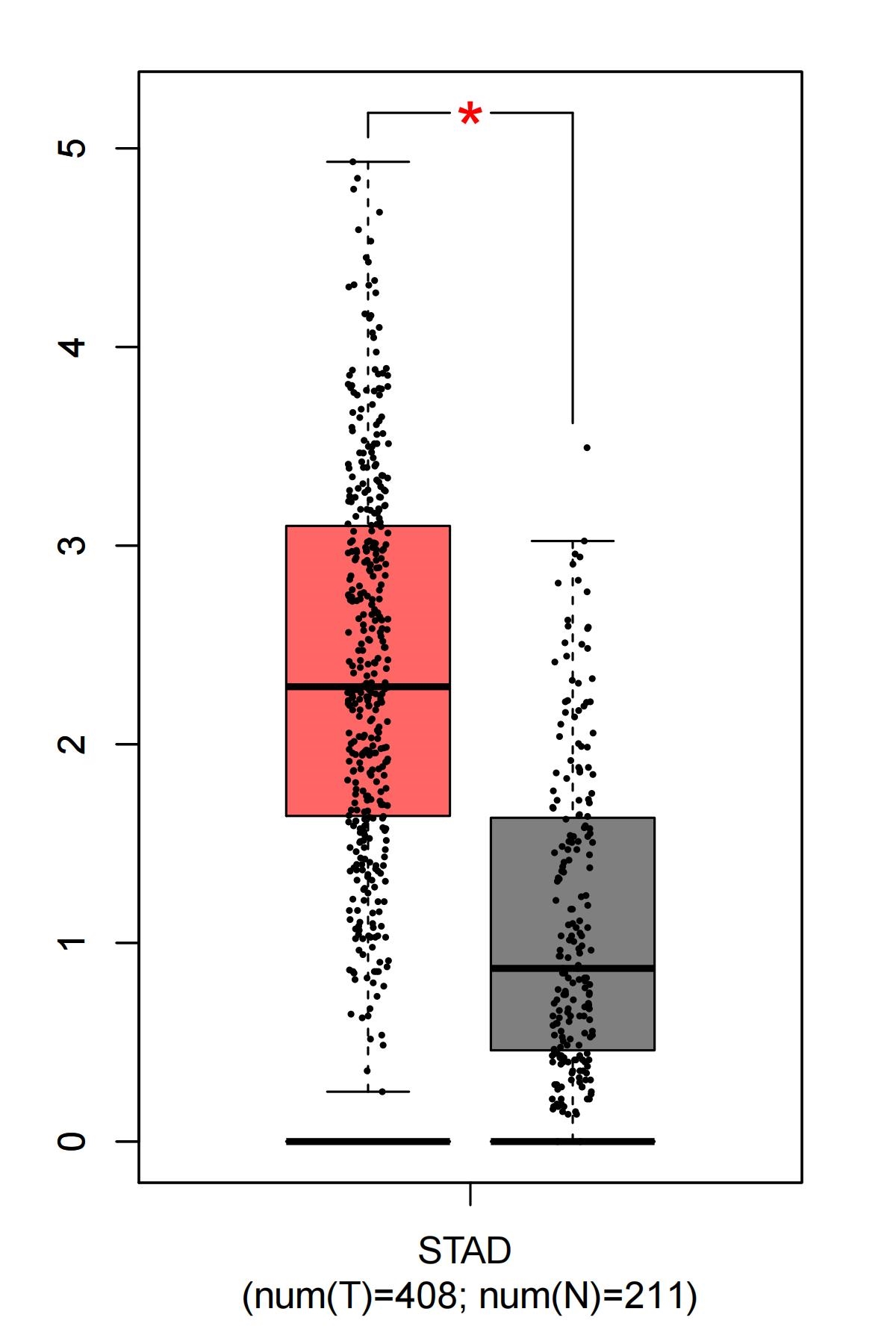

Supplement: Supplementary Figure 5 — Boxplot of differential expression of CCR1 in gastric cancer and normal tissues. [file Image_5.JPEG]
