# Supplementary material for: Establishment and Analysis of an Individualized Immune-Related Gene Signature for the Prognosis of Gastric Cancer
Source: Front Surg. 2022 Jan 31;9:829237. doi: 10.3389/fsurg.2022.829237 (PMC8841693; doi:10.3389/fsurg.2022.829237)
Supplement: Supplementary Table 1 — Correlation between TFs and immuneGenes. [file Table_1.DOCX]

**Supplementary Table 1** Correlation between TFs and immuneGenes

| **TF** | **immuneGene** | **cor** | ***p*value** | **Regulation** |
| --- | --- | --- | --- | --- |
| CBX3 | AHNAK | -0.40793 | 7.99E-15 | negative |
| CBX7 | SLC22A17 | 0.440459 | 2.77E-17 | postive |
| CBX7 | CSRP1 | 0.617326 | 1.81E-36 | postive |
| CBX7 | TPM2 | 0.55382 | 3.06E-28 | postive |
| CBX7 | AHNAK | 0.469427 | 1.05E-19 | postive |
| CBX7 | FGF2 | 0.484731 | 4.39E-21 | postive |
| CBX7 | DES | 0.663486 | 9.75E-44 | postive |
| CBX7 | KCNH2 | 0.403567 | 1.63E-14 | postive |
| CBX7 | PTN | 0.514327 | 5.97E-24 | postive |
| CBX7 | TAC1 | 0.412573 | 3.69E-15 | postive |
| CBX7 | VIP | 0.413718 | 3.05E-15 | postive |
| CBX7 | AGTR1 | 0.456108 | 1.45E-18 | postive |
| EOMES | CXCL9 | 0.412426 | 3.79E-15 | postive |
| EOMES | PNOC | 0.431172 | 1.49E-16 | postive |
| FOXP2 | CSRP1 | 0.663677 | 9.05E-44 | postive |
| FOXP2 | TPM2 | 0.594725 | 2.49E-33 | postive |
| FOXP2 | FGF2 | 0.553839 | 3.05E-28 | postive |
| FOXP2 | DES | 0.737218 | 1.74E-58 | postive |
| FOXP2 | KCNH2 | 0.457444 | 1.12E-18 | postive |
| FOXP2 | PTN | 0.547129 | 1.79E-27 | postive |
| FOXP2 | TAC1 | 0.438972 | 3.64E-17 | postive |
| FOXP2 | AGTR1 | 0.578209 | 3.45E-31 | postive |
| FOXP3 | IFI30 | 0.407895 | 8.04E-15 | postive |
| FOXP3 | CXCL9 | 0.454548 | 1.96E-18 | postive |
| FOXP3 | CCR1 | 0.43653 | 5.68E-17 | postive |
| GATA3 | TAC1 | 0.481752 | 8.24E-21 | postive |
| KAT2B | AHNAK | 0.544396 | 3.64E-27 | postive |
| KAT2B | PTN | 0.42976 | 1.91E-16 | postive |
| LMNB1 | TAP1 | 0.425741 | 3.88E-16 | postive |
| MAF | PDGFRB | 0.488958 | 1.78E-21 | postive |
| MEIS1 | SLC22A17 | 0.440147 | 2.94E-17 | postive |
| MEIS1 | CSRP1 | 0.594968 | 2.31E-33 | postive |
| MEIS1 | TPM2 | 0.531049 | 1.07E-25 | postive |
| MEIS1 | AHNAK | 0.404409 | 1.42E-14 | postive |
| MEIS1 | FGF2 | 0.508688 | 2.21E-23 | postive |
| MEIS1 | DES | 0.632233 | 1.11E-38 | postive |
| MEIS1 | PTN | 0.562426 | 2.97E-29 | postive |
| MEIS1 | AGTR1 | 0.491884 | 9.44E-22 | postive |
| MYH11 | SLC22A17 | 0.404743 | 1.35E-14 | postive |
| MYH11 | CSRP1 | 0.7182 | 3.13E-54 | postive |
| MYH11 | TPM2 | 0.651064 | 1.18E-41 | postive |
| MYH11 | AHNAK | 0.413934 | 2.94E-15 | postive |
| MYH11 | FGF2 | 0.527702 | 2.43E-25 | postive |
| MYH11 | DES | 0.792983 | 1.96E-73 | postive |
| MYH11 | KCNH2 | 0.478619 | 1.59E-20 | postive |
| MYH11 | PTN | 0.539453 | 1.29E-26 | postive |
| MYH11 | TAC1 | 0.447348 | 7.72E-18 | postive |
| MYH11 | VIP | 0.436061 | 6.19E-17 | postive |
| MYH11 | AGTR1 | 0.56746 | 7.37E-30 | postive |
| PBX1 | CSRP1 | 0.487242 | 2.57E-21 | postive |
| PBX1 | TPM2 | 0.438886 | 3.70E-17 | postive |
| PBX1 | AHNAK | 0.421439 | 8.19E-16 | postive |
| PBX1 | FGF2 | 0.490981 | 1.15E-21 | postive |
| PBX1 | DES | 0.519152 | 1.91E-24 | postive |
| PBX1 | KCNH2 | 0.404333 | 1.44E-14 | postive |
| PBX1 | PTN | 0.438124 | 4.25E-17 | postive |
| PBX1 | AGTR1 | 0.423882 | 5.37E-16 | postive |
| SOX17 | RBP7 | 0.465335 | 2.38E-19 | postive |
| SOX17 | PDGFRB | 0.566165 | 1.06E-29 | postive |
| SOX4 | TUBB3 | 0.468968 | 1.15E-19 | postive |
| STAT1 | IFNG | 0.507409 | 2.96E-23 | postive |
| STAT1 | TAP1 | 0.71907 | 2.04E-54 | postive |
| STAT1 | CXCL9 | 0.657966 | 8.44E-43 | postive |
| STAT1 | STAT1 | 0.949037 | 1.51E-168 | postive |
| STAT1 | BST2 | 0.459546 | 7.45E-19 | postive |
| STAT1 | CCR1 | 0.447534 | 7.45E-18 | postive |
| STAT1 | GZMB | 0.558984 | 7.62E-29 | postive |

**Supplementary Table 2** Analysis of the relationship between PDEIRGs and immune cell infiltration

| **Immune cell type** | **CCR1** | | **DES** | | **PNOC** | | **PRKCG** | | **RBP7** | | **SPP1** | | **TNFRSF12A** | | **TUBB3** | | **VIP** | |
| --- | --- | --- | --- | --- | --- | --- | --- | --- | --- | --- | --- | --- | --- | --- | --- | --- | --- | --- |
|  | **cor** | **P** | **cor** | **P** | **cor** | **P** | **cor** | **P** | **cor** | **P** | **cor** | **P** | **cor** | **P** | **cor** | **P** | **cor** | **P** |
| Purity | -0.213 | **<0.0001** | -0.141 | **0.00589** | -0.26 | **<0.0001** | -0.031 | 0.542 | -0.154 | **0.00259** | -0.07 | 0.172 | -0.05 | 0.328 | -0.006 | 0.906 | -0.165 | **0.00126** |
| B Cell | -0.393 | **<0.0001** | 0.182 | **0.00045** | 0.175 | **0.00073** | 0.019 | 0.713 | 0.087 | 0.0942 | -0.328 | **<0.0001** | -0.262 | **<0.0001** | -0.116 | **0.0265** | 0.125 | **0.0161** |
| CD8+ T Cell | 0.52 | **<0.0001** | 0.163 | **0.00162** | 0.527 | **<0.0001** | 0.183 | **0.00042** | 0.153 | **0.00324** | -0.025 | 0.625 | 0.06 | 0.25 | -0.193 | **0.000194** | 0.168 | **0.00118** |
| CD4+ T Cell | 0.216 | **<0.0001** | 0.481 | **<0.0001** | 0.517 | **<0.0001** | 0.098 | 0.0607 | 0.399 | **<0.0001** | -0.207 | **<0.0001** | -0.27 | **<0.0001** | -0.054 | 0.301 | 0.452 | **<0.0001** |
| Macrophage | 0.473 | **<0.0001** | 0.424 | **<0.0001** | 0.342 | **<0.0001** | 0.145 | **0.00516** | 0.512 | **<0.0001** | 0.184 | **0.000368** | -0.045 | 0.385 | -0.024 | 0.65 | 0.503 | **<0.0001** |
| Neutrophil | 0.727 | **<0.0001** | 0.094 | 0.0691 | 0.461 | **<0.0001** | 0.0117 | **0.0238** | 0.147 | **0.00454** | 0.248 | **<0.0001** | 0.097 | 0.0628 | -0.186 | **0.000309** | 0.176 | **0.000646** |
| Dendritic Cell | 0.779 | **<0.0001** | 0.283 | **<0.0001** | 0.599 | **<0.0001** | 0.193 | **0.00018** | 0.289 | **<0.0001** | 0.261 | **<0.0001** | 0.03 | 0.567 | -0.127 | **0.0144** | 0.332 | **<0.0001** |

Bold indicates that the result is meaningful.
